# Literary Notes

**Published:** 1897-10

**Authors:** 


### Literary Notes.

THE *Western Medical and Surgical Gazette* is the name of a new monthly journal of medicine and surgery established at Denver, Col., the first number of which will bear date October, 1897. It is edited by Drs. William N. Beggs and Lincoln Mussey. Each number will contain seventy-two pages of reading-matter, and a long list of collaborate and department editors is announced. A medical magazine conducted on the lines foreshadowed in the prospectus of this one deserves to succeed and is entitled to the good wishes of its contemporaries.

---

THE *Alabama Medical and Surgical Age* has been removed from Anniston to Birmingham, where it will be conducted at 2107 Third avenue under the same management as heretofore. The editor, Dr. John C. LeGrand, has established the *Age* on solid grounds and we believe the change of location is wise. Birmingham is one of the most important manufacturing cities in the south and is destined to become a metropolis.

---

The *Canadian Practitioner*, the *Dominion Medical Monthly* and the *Canadian Journal of Medicine and Surgery*, all published at Toronto, devoted their September editions to publishing the proceedings in abstract of the British Medical Association. Some of the illustrations in these journals are excellent and all their work indicates an enterprising spirit that is to be commended.

---

Messrs. William Wood & Company, medical publishers, New York, announce the early issue of the following-named books: Text-book of nervous diseases (fourth edition), by Dr. Charles L. Dana; Treatise on gynecology, clinical and operative, by S. Pozzi, M. D., Paris. The latter has been translated and printed under the personal supervision of Dr. Brooks H. Wells, editor of the *American Journal of Obstetrics.*

---

Dr. Montgomery A. Crockett, of Buffalo, contributed an article on Medical teaching to the September edition of the *Educational Review.* It is at once a critique on the curriculum and the didactic lectures of the present day. Dr. Crockett very justly asserts that we must look to the medical departments of the large universities for the first steps toward improved teaching, since it is only such schools that can offer inducements for the proper preparation of teachers. Dr. Crockett's article will well repay reading to any one interested in the advancement of medical education.

